# Cadmium and Mercury phytostabilization from soil using *Miscanthus* × *giganteus*

**DOI:** 10.1038/s41598-020-63488-5

**Published:** 2020-04-21

**Authors:** Zeljka Zgorelec, Nikola Bilandzija, Kristina Knez, Marija Galic, Silva Zuzul

**Affiliations:** 1 University of Zagreb Faculty of Agriculture, Agroecology Unit, Svetosimunska c. 25, 10000 Zagreb, Croatia; 2 University of Zagreb Faculty of Agriculture, Agricultural Engineering and Technology Unit, Svetosimunska c. 25, 10000 Zagreb, Croatia; 3 Institute for Medical Research and Occupational Health, Ksaverska c. 2, 10000 Zagreb, Croatia

**Keywords:** Element cycles, Pollution remediation

## Abstract

The determination of the effects of cadmium and mercury on the growth, biomass productivity and phytoremediation potential of *Miscanthus* × *giganteus* (MxG) grown on contaminated soil was the main aim of this paper. The use of bioenergy plants as an innovative strategy in phytotechnology gives additional benefits, including mitigation and adaptation to climate change, and soil remediation without affecting soil fertility. An experiment was set up as a randomized complete block design with the treatments varied in concentrations of Cd (0, 10 and 100 mg kg^−1^ soil) and Hg (0, 2 and 20 mg kg^−1^ soil) added to the soil. Three vegetative years were studied. Yield values ranged from 6.3–15.5 t_DM_ ha^−1^, cadmium concentration in plants varied from 45–6758 µg kg^−1^ and Hg varied from 8.7–108.9 µg kg^−1^. Values between treatments and years were significantly different. MxG can accumulate and remove very modest amount (up to 293.8 µg Cd and 4.7 µg Hg) per pot per year in aboveground biomass. Based on this data it can be concluded that MxG, as a valuable energy crop, is a potential candidate for the phytostabilization and biomass production on soils contaminated with Cd and Hg moderately.

## Introduction

Phytoremediation is considered as a simple and a natural technology that uses plants which can be utilize for efficient absorption of pollutants from contaminated soils^1,2^. Generally, remediation of heavy metal polluted soils could be classified as physico-chemical and biological remediation techniques^3^. Compared to physico-chemical techniques (vitrification, soil washing, solidification and stabilization), phytoremediation technology could reduce dust emission, risk of exposure to soil, provide erosion control and prevent runoff^4^. Unlike physical and chemical treatments that irreversibly alter soil properties, phytoremediation generally improves physical, chemical, and biological quality of contaminated soils, improving soil quality and functionality and carbon sequestration^5,6^. Phytoremediation is suitable for different types of contaminants (organic, metals and radionuclides), with relatively low financial costs, does not require additional energy delivery (energy is obtained from solar radiation) and with minimally influence to the site destruction and destabilization. Additionally, it contributes to the improvement of the visual aspect of the landscape, provides habitats for animals, and reduces leaching and mobilization of contaminants in soil^7^. Disadvantages of phytoremediation include: long remediation time requirement (the process is slow and requires 3–20 growing seasons to achieve remediation goals); relatively shallow cleaning depths; potentially contamination of the food chain; a site-specific technology (structure of the soil profile, pH, presence of salt or other toxins, water quality including nutrients, oxygen content) with a choice of plants being critical, and the fate of contaminants often unclear (the technology may relocate contaminants from the subsurface to the plant, creating residual waste to be disposed of); groundwater contamination possibility and climate dependence^4,6–8^. The technology is applicable only to moderately contaminated land, it cannot compete with conventional remediation on heavily polluted sites. There is also concern about the content of toxic elements in the biomass of energy crops produced on a contaminated land that may generate hazardous emissions^7^. Based on removal mechanisms and type of pollutants, phytoremediation techniques can be categorized as phytoextraction, phytovolatization, phytofiltration, phytostabilisation, phytodegradation and rhyzophere bioremediation^9^. Phytoremediation is technique that can be used for the reduction of bioavailability and mobility of heavy metals in soils^1,2,10,11^ and is defined as possibility of plants to increase retention of specific metals in root zone^12^. Phytoextraction and phytostabilization are the most wide used remediation techniques, and differ in accumulation of elements in above ground or root parts of plants. Generally, advantages of phytostablisation are effective rapid immobilization and no need for biomass disposal, while major disadvantage is a fact that pollutants still remain in the soil^13^ or in the root system, generally in the rhizosphere. In comparison to phytoextraction, where pollutants are accumulated in the biomass, which is consider a possible problem afterwards. Phytostabilisation has proved to be useful for the treatment of Pb, As, Cd, Cr, Cu and Zn contaminated soils^9^. Phytoremediation is a very complex biotechnology, which is under the significant influence of (I) plant morphology (growth rate, biomass yields) and physiology (accumulation potential, stress tolerance)^14,15^, (II) agro-ecological conditions of cultivated land (soil type and environment)^16–18^, (III) agronomy practices (cultivars, planting density, soil amendments application)^19–21^, (IV) origin of contamination^22–24^. To increase the potential of phytoremediation, which is usually limited by low above ground biomass and/or a shallow root system^25–27^ more and more attention has been given to perennial plants with high biomass yield potential^20,28^. The use of fast growing energy crops for purifying polluted lands is an innovative strategy to derive additional benefits from such remediation activities^29–31^, it may have important role from ecological and energy point of view^7^. Due to the competition for arable land, water and nutrient resources, an implementation of energy crops in the phytoremediation strategy directly avoids the potential conflict between food and fuel production^31–33^. The potential soil amendments and phytoremediation stimulants, including (I) mineral fertilizers^28^, (II) farm manure^34^, (III) organic wastes/biosolids (sewage sludge, compost)^35,36^, (IV) solid bioproducts (biochar)^19^, (V) organic substances/biostimulants (mycorrhizal fungi)^21^ can be used to increase the biomass yield, the absorption potential of the plants, the amount of soil organic matter and to immobilize the metals in soil. Due to its morphological and physiological characteristics, one of the most investigated bioenergy plant for the purposes of remediation is *Miscanthus* × *giganteus* (MxG). Soils that have suffered from physical, biological and/or chemical degradation (i.e. soils contaminated with trace elements), or are uncultivated or/and adversely affected by climate conditions could be defined as marginal lands^37^. Elbersen *et al*. mapped 29% of agricultural land in EU being marginal^38^. Production of large quantities of biomass, thus providing the effective phytoremediation showed good potential of using *Miscanthus sp*. commercially on marginal sites in the regions of Central and Eastern Europe, and United States^39,40^. Khalid reported that the most efficient remediation could be achieved with high biomass plants utilization^11^. Nsanganwimana *et al*. highlighted MxG as a promising crop for the phytostabilization^21^, while Kerr characterized it as a tolerant phytoremediator in terms of growth on heavily polluted soils contaminated with Cu, Cd, Cr, Pb, Ni and Zn^41^. Fernando and Oliveira investigated the influence of heavy metals (Cd, Cu, Cr, Hg, Ni, Pb and Zn) on plant growth and productivity of MxG^42^. Although above mentioned authors recommend application of MxG plant species in phytoremediation, the higher heavy metal concentrations could negatively affect plant growth and productivity. Barbosa *et al*. noted that Zn contamination only reduced significantly MxG production, while not *M. sinensis* or M. *floridulus* yields and concluded that *Miscanthus* genotypes have shown different phytoremediation potential^43,44^. Some authors^21,40,45,46^ classify MxG as excluder. Barbu *et al*. investigated the possibility of using MxG for Cd uptake and reported accumulation of 35–55 g Cd per hectare per year^47^, and Nsanganwimana *et al*. revealed that majority of Cd accumulation was in roots of the plant^21^. Furthermore, on soils contaminated with 0.01 g Cd m^−2^, *Miscanthus* accumulated 0.013 mg kg^−1^ in its shoots^42^. Rosikon *et al*. observed positive influence of sewage sludge on Cd bioaviability by MxG^20^ by investigating the influence of sewage sludge fertilization applied at different rates to *M. sacchariflorus* and compared with plant treatment by mineral fertilizers for uptake of different metals into stems and leaves during two years of observation. Cadmium was not detected in *M. sacchariflorus* biomass in the first year whereas large amounts of the metal were recorded in the second year (6–9 mg kg^−1^)^39,48–50^. The increased accumulation of Cd in MxG shoots with increasing Cd concentrations in the soil induced a reduction in plant height and shoot dry weight^40^. *Miscanthus* showed low tolerance to Hg toxicity in terms of biomass productivity. It can be grown in fields contaminated with Hg only for soil remediation purposes, since economically might not be feasible due to decreased productivity. Effectively, with the increased Hg concentrations in the soil, biomass showed significantly higher accumulation of Hg, with lower biomass production, in comparison with the control^49^. The average typical value of Cd in *Miscanthus* crops was reported 0.1 mg kg^−1^ and for Hg was 0.03 mg kg^−1^ and for grass in general 0.2 mg Cd kg^−1^ and <0.02 mg Hg kg^−1^ ^51^. The main aim of this paper was to determine the effects of cadmium and mercury on the growth, biomass productivity and phytoremediation potential of MxG grown on contaminated soil.

## Materials and Methods

### Experimental plot

The experiment was set up on 4^th^ of March in 2014, in an open greenhouse in plastic experimental pots (EP). Weather conditions (sunlight duration, temperature and precipitation) were natural and soil moisture was controlled on daily basis and if necessary maintained to the field water capacity. According to the data of Croatian Meteorological and Hydrological Service (station Maksimir, Zagreb) mean annual values of precipitation (887.1 mm; 858.6 mm and 897.0 mm) and temperatures (12.6 °C; 12.2 °C and 12.6 °C) were noted for 3 studied vegetative years (2015, 2016 and 2017). The experiment was set up according to the completely randomized design and MxG was planted in four treatments in three replications during three years. High quality rhizomes (15 × 15 × 15 cm, cube of plant/soil) of three-year-old *Miscanthus* from Bistra field in the Republic of Croatia were planted in truncated cone plastic pot EP (Ø_T_ = 28 cm; Ø_B_ = 19 cm; h = 29 cm;) where Ø_T_ and Ø_B_ present a top and bottom diameter of EP, respectively.

### Preparation of contaminated soil

Four treatments (C, L_1_, L_1_ + SS, L_2_) varied in concentration levels of Cd (0, 10 and 100 mg kg^−1^) and Hg (0, 2 and 20 mg kg^−1^) were applied to the soil. The first control group (C) consisted of pure soil. The soil in the second group was treated with lower level (L_1_) of contaminants: 10 mg Cd kg^−1^ (in CdO (s) form) and 2 mg Hg kg^−1^ (in HgCl_2_ (s) form). A third group (L_1_ + SS) was treated with identical concentrations of Cd and Hg applied to soil as in L_1_, but with an addition of sewage sludge in an equivalent of maximal 1.66 t_DM_ ha^−1^ according to Croatian legislative^52^. Soil in the fourth group was treated with a higher level (L_2_) of contaminants: 100 mg Cd kg^−1^ and 20 mg Hg kg^−1^ of soil. Contaminants were applied as p.a. salts in solid phase to dry soil before first vegetative year. Subsamples of clean soil were mixed with adequate amounts of salts to achieve homogeneity and then were vigorously mixed for a long time with the whole pot volume mass (~18 kg).

### Soil characteristics

Soil used in the experiment was characterized as silt-loam texture (66.3% silt, 21.3% sand and 12.4% clay; sieving and sedimentation method were used^53^) with acid reaction (pH_KCl_ = 5.12; obtained in 1 M KCl in 1:2.5 (*m/v*)^54^) and having low content of organic matter (OM = 2.26%; determined by wet combustion method with sulfochromic oxidation^55^). Soil was classified as Stagnosol^56^. Soil was well supplied with total nitrogen (0.12%, determined by dry combustion (Dumas) method^57^). All light elements (C, H, N and S) were analyzed by dry combustion method on Vario Macro CHNS analyzer, Elementar, 2006. Soil was low supply with plant available potassium (74 mg kg^−1^) and phosphorous (26 mg kg^−1^); (AL method; extraction with ammonium lactate acetic acid in 1:20 (*m/v*) ratio^58^). CEC was 18.4 cmol^+^ kg^−1^ (determined using barium chloride method in 1:40 (*m/v*) ratio^59^). Total Cd and Hg in soil were measured in aqua regia extract^60^ on AAS equipped with graphite and hydride technique (SOLAR AA Spectrometer M Series, Thermo Scientific, 2008 with Graphite Furnace and Cold Vapour System; see plant analysis, Table 1). Measured Cd concentration was 119 μg kg^−1^, which was far below MAC (maximal allowable concentration) for agricultural soils (MAC = 1500 µg Cd kg^−1^ for soils with pH value between 5 and 6) and measured Hg concentration was 66 μg kg^−1^, which was also far below MAC value for agricultural soils (MAC = 1000 µg Hg kg^−1^) according to Croatian legislative^61^.

**Table 1:** Parameters and methods used in biomass analysis.

Parameter	Protocol/Norm
Drying/grinding/milling/homogenizing	HRN ISO 11464:2009^80^ at 60 °C to constant mass -> plant powder
w(ST), w(H_2_O) [%]	HRN ISO 11465:2004^62^
Cd and Hg extraction	HNO_3_:H_2_O_2_ = 5:1 (*v/v*) digestion in ratio 1:30 (*m/v*) 0.2 g -> 6 mL -> 50 mL
Cd [μg kg^−1^]	HRN ISO 11047:2004^81^ ISO/TS 16965:2013^82^
Hg [μg kg^−1^]	HRN ISO 16772:2009^83^

### Sewage sludge characteristics

Wastewater sewage sludge (SS) was characterized having neutral pH value (pH_KCl_ = 7.54 in 1 M KCl in 1:2.5 (*m/v*) ratio^54^) and 63% of water^62^. Total carbon content was 22.7% (determined by dry combustion^63^). Content of hydrogen was 9.52%, nitrogen 2.24%^57^ and sulphur 0.36%^64^. A total phosphorous content was 1.35% (extraction in aqua regia^60^; and detection by ICP-OES^65^, ICP-OES, Vista MPX Axial, Varian, 2004). The concentration of total cadmium in municipal waste water sewage sludge was 349 μg kg^−1^ which was approximately 3 times higher than in soil, and concentration of mercury was 299 μg kg^−1^, which was 4.5 times higher than in soil. Still, concentrations of Cd and Hg in sewage sludge were far below the permitted content of heavy metals in the sludge prescribed by Croatian law while used in agriculture (5000 μg kg^−1^ for Cd and Hg^52^).

### Biomass sampling and growth parameters

The sampling of MxG was conducted at the beginning of March in 2015, 2016 and 2017 for each experimental pot. The biomass harvest was carried out by manual cutting of the plants at the height of 5 cm from the soil. Whole above ground biomass represents the sample with all dead leaves which were collected, if were any. Yield parameters, including plant height, shoot numbers per rhizome and mass of biomass with natural moisture content were determined on the site. Afterwards samples were cut to smaller pieces and transported to the Lab. The dry matter yield was determined gravimetrically after drying at 60 °C to the constant mass. Afterwards samples were milled to a powder and proceeded to digestion and metals analysis.

### Biomass analysis

Table 1 shows methods used in the analysis of biomass. Aliquots of dried and homogenized plants (about 0.2 g) were wet digested with combination of HNO_3_ and H_2_O_2_ (5:1 = (*v/v*)). All chemicals used in digestion were high purity (p.a.). Wet digestion was conducted in the digestion block (Velp, 2007) using the programme for Cd, 1 h at 65 °C and 3 h at 150 °C and for Hg, 4 h at 55 °C. The digest was diluted up to 50 mL with Milli-Q water. Mercury and cadmium detection in plants harvested in 2015 were determined with AAS (SOLAR AA Spectrometer M Series, Thermo Scientific, Autosampler, 2008). For Cd detection, graphite technique was used (Graphite Furnace, GF 95 + FS % Furnace) and for Hg detection hydride technique was used (Vapour System, VP100). In plants harvested in 2016 and 2017, Hg concentrations were detected with a PerkinElmer Flow Injection Mercury Hydride System, FIMS 400 with autosampler AS-91, 2006 and Cd concentrations (isotope 111) were determined by inductively coupled plasma mass spectrometry (ICP-MS 7500 cx, Agilent Technologies, Waldbronn, Germany) with rhodium as internal standard. The ICP-MS was tuned so that the oxides and doubly charged ions were less than 2% and each solution was analyzed in triplicate, in full quant mode, with helium as collision gas.

### Statistical analysis and quality control

Statistical analysis was done with the use of statistical software SAS 9.1 (SAS Inst. Inc.), One-Way ANOVA and post-hoc (Fisher LSD) test were used for processing of data. The threshold of significance was 5% for all tests. Quality control was included. Measurement accuracy and method precision for Cd and Hg determination were checked using reference materials (IPE 171 and IPE 186 for plant and ISE 865 for soil, Wageningen University) and were satisfactory. Absolute error for Cd measurements was up to maximal 8% and for Hg up to 5%, respectively. Relative standard deviation (RSD) or repeatability of measurement for Cd was up to maximum 7% and for Hg 8%, respectively.

## Results and Discussion

### Growth parameters

**Figure 1:**
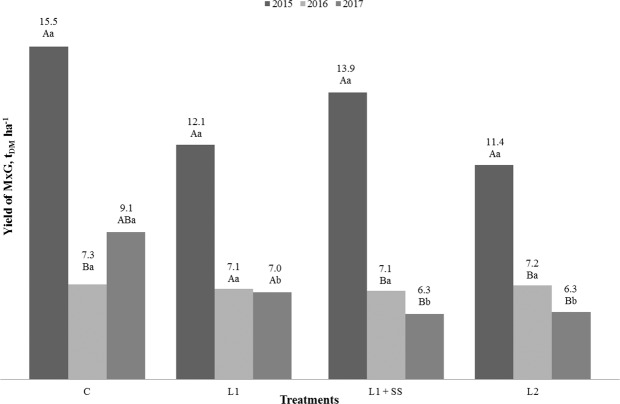
Yield of MxG (t_DM_ ha^−1^) according to treatments and studied years. Mean values marked with the same capital letters between the years and the same small letters between treatment are not statistically significant (Fisher test).

**Figure 2:**
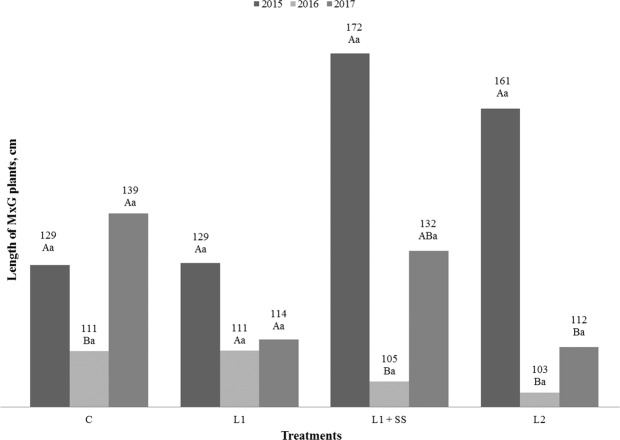
Length of MxG plants according to treatments and studied years. Mean values marked with the same capital letters between the years and the same small letters between treatments are not statistically significant (Fisher test).

**Figure 3:**
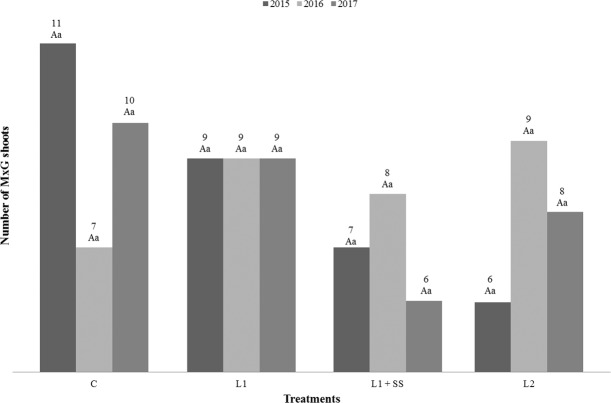
Number of MxG shoots according to treatments and studied years. Mean values marked with the same capital letters between the years and the same small letters between treatments are not statistically significant (Fisher test).

Results of the study including yield, length of plants, number of shoots regarding the treatment options and vegetative years are presented in Figs. 1 to 3. Statistical analysis of the results shows the influence of vegetative years on the yield for MxG by different treatment. The significant difference for C, L_1_ +SS and L_2_ treatments has been determined between the years of investigation (Fig. 1). In comparison to the first year of the study, the decrease of the MxG’s yield was found in the second and third year, both on the control treatment and on the contaminated soil, in the range of 37% up to 55%. It is highly unlike that the shoots will accumulate any significant amount of heavy metals from the soil in the first year of growth and have a significant impact on the yield, thus obtained values were expected. However, only in the control treatment the increase in the yield was observed in the third year. An interaction between MxG yield and treatments displays the statistically significant difference only in the third year of the research. It can be noticed that the highest yield was determined in the control treatment C (7.3–15.5 t_DM_ ha^−1^), and the lowest in the treatment L_2_ (6.3–11.4 t_DM_ ha^−1^). Even application of municipal sewage sludge, as a soil amendment was not resulting in biomass increase because the yield on sewage sludge treatment compared to L_1_ was not statistically significant. Thus, no impact on soils slightly contaminated with Cd and Hg during three-year investigation period was observed. The negative impact of Cd and/or Hg on biomass yield was also determined by Arduini *et al*. and Fernando and Oliveira^42,45^. Antonkiewicz *et al*. found a positive impact of sewage sludge on the yield, in five years long research^66^. However, they applied up to 36 times higher doses of municipal sewage sludge (0–60 t_DM_ ha^−1^) in comparison to our study. The average yield in their study was reported 15.3 t_DM_ ha^−1^ in control treatment and 16.6 t_DM_ ha^−1^ in treatment with sewage sludge, respectively. The length of the plant was not statistically influenced by the treatments, while significant differences can be seen between years of research for treatments C, L_1_ + SS and L_2_ (Fig. 2). If we compare first and third year, the increasing length of the plant in treatment C could be noticed; while this was not the case for other treatments. Length of the plant in this study ranged from 103 cm up to 172 cm. Fernando and Oliveira, and Arduini *et al*. determined the reduction of plant length in relation to the increase of Cd concentration in the soil^42,67^. Zhang *et al*. noted that growth of *Miscanthus sacchariflorus* was significantly inhibited when Cd concentration in the soil was above 50 mg kg^−1^ compared with control^68^. Kocoń and Jurga were investigating shoot numbers and shoot length of MxG and *Sida hermaphrodita* on two different soil textures, including sandy and loamy soil contaminated with Cd, Cu, Ni, Pb and Zn of the second year of cultivation. They determined that MxG had greater number of shoots and lower shoot length compared to *Sida hermaphrodita* regardless of soil texture^28^. However, Fernando and Oliveira did not observe the negative impact of Hg on the length of MxG plant^42^. There were no statistically significant differences between treatments and years for the number of MxG shoots (Fig. 3). The number of shoots per plant in this research ranged from 6 up to 11 and these observed values are lower compared to research conducted by Arduini *et al*. and Pogrzeba *et al.*^45,69^. Like with plant height, Fernando and Oliveira observed that treatments with Hg in the soil did not have an impact on the number of shoots of MxG^42^.

### Cadmium and mercury concentration in plants

**Figure 4:**
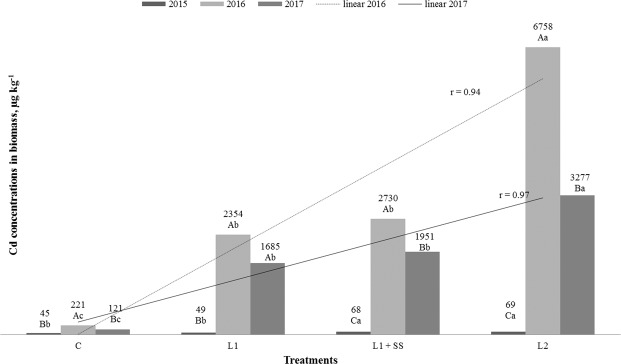
Cd concentrations in MxG according to treatments and studied years. Mean values marked with the same capital letters between the years and the same small letters between treatments are not statistically significant (Fisher test).

**Figure 5:**
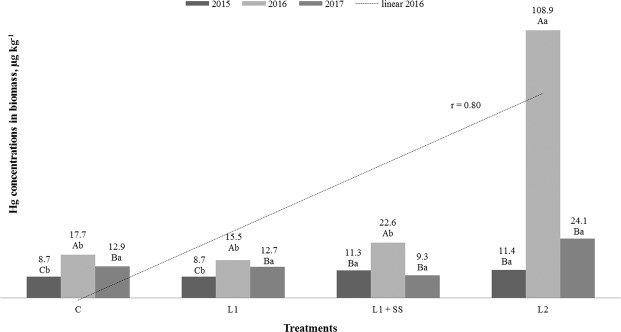
Hg concentrations in MxG according to treatments and studied years. Mean values marked with the same capital letters between the years and the same small letters between treatments are not statistically significant (Fisher test).

Concentrations of cadmium and mercury in the aboveground biomass of MxG, in relation to treatments and years of research are shown in Figs. 4 and 5. Statistically significant influence of years and treatments on concentration of Cd and Hg in aboveground biomass is revealed. Cadmium concentration in plants varied from 45 µg kg^−1^ (C, 2015) up to 6758 µg kg^−1^ (L_2_, 2016**)**, and Hg varied from 8.7 µg kg^−1^ (C and L_1_) measured in 2015 up to 108.9 µg kg^−1^ (L_2_**)** observed 2016 (Figs. 4 and 5). Values of Cd in the first investigated year were low in all treatments, much lower than typical value (100 μg kg^−1^) of Cd in *Miscanthus* according to HRN ISO 17225-1:2014^51^ (Solid Biofuels-Fuel specifications and Classes-Part 1: General requirements) while, in the second and third year of investigation the concentration of Cd in MxG were higher in C and much higher in all other contaminated treatments. In the second and third year of investigation, significantly higher concentrations of Cd in MxG are observed regarding rising doses of Cd in the soil, and correlation are completely positive^70^ (Fig. 4), while, according to Pogrzeba *et al*.^69^ Cd concentration where at similar levels during all three years of investigation. Arduini *et al*. investigated Cd in MxG stems, in relation to different concentrations of Cd in soil (0.75–3.00 mg L^−1^), and revealed 275 up to 1237 μg of Cd per plant^45^. In relation to *Sida hermaphrodita*, the MxG accumulate better Zn, Cu, Pb, Ni compared to Cd^39^. Antonkiewicz *et al*. studied an application of different sludge doses (0–60 t_DM_ ha^−1^) to the soil and reported the average (5 years) concentration of Cd in aboveground biomass from 90 to 180 μg kg^−1^ ^66^. Zhang *et al*. measured Cd concentration in *M. sacchariflorus* in aboveground part of 0.92, 2.28, 4.41, 6.26 and 18.36 mg kg^−1^ on soil contaminated with 1, 5, 25, 50 and 100 mg Cd kg^−1^, respectively^68^. According to Rosikon *et al*., different fertilize treatments have positive influence on Cd accumulation by the MxG and negative by the *Phalaris arundinacea* L. Furthermore, same authors observed that MxG and *Phalaris arundinacea* L. have lower possibility of Cd accumulation in the second year of cultivation compared to the first year, what is opposite to the results of our investigation^20^. The same as, for Cd, the highest concentrations of Hg in the MxG have been determined in the second year of research (Fig. 5). Statistically significant differences of Hg in the MxG are observed between years. For the first and second year of investigation, we noted statistically significant differences of Hg in the MxG between treatments too. All measured values of Hg in the MxG except those revealed at L_2_, 2016 (108.9 μg kg^−1^) were below typical (30 μg kg^−1^) of Hg in the MxG according to HRN ISO 17225-1:2014^51^. Fernando and Oliveira investigated Hg concentration in the MxG aboveground biomass, cultivated on soils with two different levels of contamination (5000 and 6700 μg Hg m^−2^), and determined that contamination with lower Hg dose resulted with Hg in biomass below limit of detection of method (<LOD), while contamination of soil with higher Hg dose resulted with Hg concentration in the biomass of 4 μg kg^−1^ ^42^. Pérez-Sanz *et al*. investigated mercury uptake by *Silene vulgaris*, grown on contaminated (5.5 mg Hg kg^−1^) spiked soils (alkali and neutral pH) and observed that *S. vulgaris* retains more Hg in the root (3700 and 2900 μg kg^−1^) than in aerial part (550 and 980 μg kg^−1^)^71^. Still, plants grew healthy and showed good appearance throughout the study without significantly decrease in the biomass production. Hg values in the aboveground biomass of the MxG in this study are considerably lower than those expected. However, it is not surprising due to Hg as a specific element and its behavior being a bit different than all other heavy metals. Mercury has been known as an environmental pollutant for over a century and it is well known that it may evaporate (volatilization) from some compounds and be released to various ecosystems. When added to the soil, whether in elemental, inorganic or organic form, it is likely to be strongly bond. Generally, 97–99% of total Hg is in complex form, and behavior of Hg species in the soil is controlled by soil factors, especially temperature, pH, texture, organic matter content but also the concentration of all other ions. Phytoavailability and toxicity of Hg in the soil-plant system depend on the forms in soil^72^. Lomonte *et al*. study has shown that Hg accumulated by *C. zizanioides* via root uptake is mainly present in the root epidermis and exodermis and its translocation to the aerial parts is insignificant^73^. In contrast, another study done by Lomonte *et al*. shows that some species (*Atriplex conodocarpa* and *Australodanthonia caespitose*) can be good candidates for mercury phytoextraction because of their ability to translocate mercury from roots to the aboveground tissues^74^. Lomonte *et al*. also study Hg behavior in soils and Hg efflux to atmosphere^75^. They applied biosolids (3.5–8.4 mg Hg kg^−1^) from waste water treatment plant to soil and investigated potential for Hg remediation. They observed that 59% of the total mercury was complexed with organic ligands and that the influence of water content and irradiation on the emission of gaseous elemental mercury are the main factors affecting this emission with flux values up to 132 ngm^−2^ h^−1^. Lomonte *et al*. revealed that some ions mobilize Hg in the soil, creating chelate-assisted phytoextraction for some species and increase its uptake in the plant shoots^76^. Pogrzeba *et al*. also studied Hg behavior in contaminated soil with addition of granular sulphur and observed that in Hg stressed environment, plant (*Poa pratensis*) developed the defense mechanism resulting in the reduction of Hg evaporation and higher S content in plant tissue^77^. Those authors recommended this technology for soil remediation heavily contaminated with mercury.

### Cadmium and mercury biomass removal

**Figure 6:**
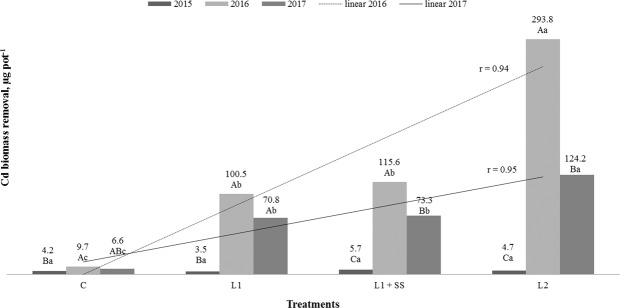
Cd biomass removals by MxG according to treatments and studied years. Mean values marked with the same capital letters between the years and the same small letters between treatments are not statistically significant (Fisher test).

**Figure 7:**
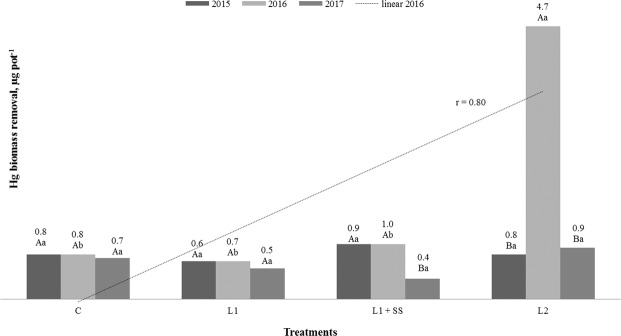
Hg biomass removals by MxG according to treatments and studied years. Mean values marked with the same capital letters between the years and the same small letters between treatments are not statistically significant (Fisher test).

Cadmium and Hg biomass removals by MxG according to treatments and studied years are presented in Figs. 6 and 7. Statistically significant interaction between years of research and Cd removal is determined for all treatments, with the highest biomass removal determined in the second year. In the first year of investigation, Cd biomass removal between the treatments is not statistically significant and values determined below 10 µg pot^−1^. In the second and third year of investigation, significant increase in Cd biomass removal is noted and is in a complete positive correlation with Cd concentration in soil (Fig. 6)^70^. Values of Cd removal ranged from 70.8 up to 293.8 µg per pot (11.8–49.0 g ha^−1^) observed on contaminated treatments (L_1_ and L_2_), in the second and third year of investigation, respectively. This is in accordance to Barbu *et al*. who determined an uptake of 35–55 g Cd ha^−1^ ^47^. Bang *et al*. noted limited Cd accumulation by *Miscanthus* in a marginally contaminated ecosystem, although they observed 100% of Cd removal from contaminated water after 40 days^78^. Yao *et al*. observed Cd accumulation in plant tissue of 2.2 mg Cd m^−2^ (22 g ha^−1^) in *Miscanthus sacchariflorus* and 700 kg Cd per year where it was accumulated by aboveground organs and removed from the lake (Dongting Lake wetlands, China) through harvesting for paper manufacture^79^. Hg biomass removals by the MxG according to treatments and studied years varied up to 4.7 µg per pot (0.79 g ha^−1^) in our study. Statistically significant influence of years on Hg removal has been determined for treatments L_1_ + SS and L_2_. In terms of interaction between treatments, significant Hg removal is observed only in the second year of research in treatment L_2_ (Fig. 7).

## Conclusion

Values of Cd in the MxG in first investigated year were low in all treatments, much lower than the typical value when MxG is used as a biofuel (100 μg kg^−1^). The significantly higher concentrations of Cd in the MxG were observed in the second and third year of investigation due to increased doses of Cd in the soil, and correlations were completely positive.

The detected mercury concentration in MxG plants was very low. The whole measured values of Hg in MxG except those of treatment L_2_, 2016 (108.9 μg kg^−1^) were below typical for MxG used as biofuel (30 μg kg^1^).

Finally, it can be concluded that MxG, as a valuable energy crop, could be a good candidate for the Hg and Cd phytostabilization, due to the low metal accumulation in aboveground biomass. This could be benefit for biomass production of MxG on soils moderately contaminated with Cd and Hg, where contamination still not significantly affected the yields amounts.

Except phytostabilization of the contamination, the MxG can also be used in locations where some other remediation strategies of ecosystem/agroecosystem need to be achieved, like prevention of soil erosion due to the high biomass above but also below ground.

## Data Availability

The row datasets generated and/or analyzed during this study are available from the corresponding authors upon request.
